# Impulsivity, Self-control, Interpersonal Influences, and Maladaptive Cognitions as Factors of Internet Gaming Disorder Among Adolescents in China: Cross-sectional Mediation Study

**DOI:** 10.2196/26810

**Published:** 2021-10-27

**Authors:** Yanqiu Yu, Phoenix Kit-Han Mo, Jianxin Zhang, Jibin Li, Joseph Tak-Fai Lau

**Affiliations:** 1 Center for Health Behaviours Research Jockey Club School of Public Health and Primary Care The Chinese University of Hong Kong Hong Kong Hong Kong; 2 West China School of Public Health Sichuan University Chengdu China; 3 Department of Clinical Research State Key Laboratory of Oncology in South China, Collaborative Innovation Center for Cancer Medicine Sun Yat-sen University Cancer Center Guangzhou China

**Keywords:** cognition, adolescent health, health risk behaviors, internet, self-control, China

## Abstract

**Background:**

Gaming disorder, including internet gaming disorder (IGD), was recently defined by the World Health Organization as a mental disease in the 11th Revision of the International Classification of Diseases (ICD-11). Thus, reducing IGD is warranted. Maladaptive cognitions related to internet gaming (MCIG) have been associated with IGD, while impulsivity, self-control, parental influences, and peer influences are key risk factors of IGD. Previous literature suggests that MCIG is associated with the aforementioned 4 risk factors and IGD, and may thus mediate between these risk factors and IGD. These potential mediations, if significant, imply that modification of MCIG may possibly alleviate these risk factors’ harmful impacts on increasing IGD. These mediation hypotheses were tested in this study for the first time.

**Objective:**

This study tested the mediation effects of MCIG between intrapersonal factors (impulsivity and self-control) and IGD, and between interpersonal factors (parental influences and peer influences) and IGD among adolescents in China.

**Methods:**

An anonymous, cross-sectional, and self-administered survey was conducted among secondary school students in classroom settings in Guangzhou and Chengdu, China. All grade 7 to 9 students (7 to 9 years of formal education) of 7 secondary schools were invited to join the study, and 3087 completed the survey. The *Diagnostic and Statistical Manual of Mental Disorders* (*DSM-5*) checklist was used to assess IGD. MCIG was assessed by using the Chinese version of the Revised Internet Gaming Cognition Scale. Impulsivity, self-control, and parental or peer influences were measured by using the motor subscale of the Barratt Impulsiveness Scale, the Brief Self-Control Scale, and the modified interpersonal influence scale, respectively. Structural equation modeling was conducted to examine the mediation effects of MCIG between these risk factors and IGD.

**Results:**

The prevalence of IGD was 13.57% (418/3081) and 17.67% (366/2071) among all participants and adolescent internet gamers, respectively. The 3 types of MCIG (perceived rewards of internet gaming, perceived urges for playing internet games, and perceived unwillingness to stop playing without completion of gaming tasks) were positively associated with IGD. Impulsivity, self-control, parental influences, and peer influences were all significantly associated with the 3 types of MCIG and IGD. The 3 types of MCIG partially mediated the associations between the studied factors and IGD (effect size of 30.0% to 37.8%).

**Conclusions:**

Impulsivity, self-control, and interpersonal influences had both direct and indirect effects via MCIG on IGD. Modifications of the 3 types of MCIG can potentially reduce the harmful impacts of impulsivity and interpersonal influences on IGD and enhance the protective effect of self-control against IGD. Future longitudinal studies are warranted.

## Introduction

After the inclusion of internet gaming disorder (IGD) in the *Diagnostic and Statistical Manual of Mental Disorders, Fifth Edition* (*DSM-5*) in 2013 [1], World Health Organization (WHO) recognized gaming disorder (online and offline) as a disease in the 11th Revision of *International Classification of Diseases* (*ICD-11*) in 2019 [2]. The reported range of prevalence of IGD among Chinese adolescents is wide (2.4% to 21.5%), possibly due to methodological differences [3]. Adolescents are vulnerable to IGD, which has many adverse consequences (eg, loneliness and depression [4-6]).

Maladaptive cognition is an important intrapersonal factor of addictive behaviors like pathological gambling [7] and internet addiction [8]; maladaptive cognitions related to internet gaming (MCIG) have also been associated with IGD [8-10]. A systematic review of 36 studies of MCIG proposed a 4-factor cognitive framework (ie, overvaluation of gaming rewards, maladaptive rules, gaming for self-esteem, and gaming for social acceptance) [11]; the 4 types of MCIG were all positively associated with IGD among adolescents [9]. A recent validation study modified this 4-factor model and revealed a new 3-factor model (ie, perceived rewards of internet gaming, perceived urges for playing internet games, and perceived unwillingness to stop playing without completion of gaming tasks) that demonstrated satisfactory psychometric properties [12]. The 3 domains of MCIG were all positively correlated with IGD [12]. The revised scale was used in this study.

Impulsivity and self-control are 2 important intrapersonal factors of addictive behaviors, including substance use [13,14], smoking [15], alcohol drinking [16], internet addiction [17], and IGD [4,18-21]. Impulsivity and self-control fit into the dual-process theories of decision-making for risk behaviors [22-25]. Impulsivity represents reactive, intuitive, and affective processes with high responsiveness to temptations and prompt actions without deliberation [24]. In contrast, self-control reflects the reasoned process and ability in regulating impulses deliberatively [26,27]. The 2 processes jointly affect decisions in the performance of risk behaviors [22-25]. Adolescents with high impulsivity may exhibit heightened spontaneous responses to behavioral cues to internet gaming, while those with low self-control may find it difficult to resist the temptation of playing internet games and stop playing.

Interpersonal influences are important factors of IGD. Empirical studies have reported positive associations between interpersonal influences (eg, parents’ invitations of playing internet games and intensity of peers’ gaming behaviors) and IGD or internet addiction among high schools students [28,29]. Interpersonal influences may affect IGD in different ways. Significant others’ direct invitations to play internet games may trigger prompt engagement in internet gaming. According to the health belief model, such invitations to play internet games represent cues to action, which is an important determinant of health-related behavior [30]. Frequent invitations may increase adolescents’ gaming intensity, which is associated with risk of IGD [28]. In addition, the reciprocal determinism construct of social cognitive theory (SCT) postulates that one’s environment, personal factors, and health-related behaviors interact with each other [31]. Having significant others playing internet games frequently forms a social environment that may increase adolescents’ gaming frequency. More importantly, SCT postulates that observational learning is an important factor of health-related behavior [31]; adolescents may play internet games frequently through observational learning from their significant others’ frequent internet gaming.

Importantly, MCIG are potential mediators of the association between impulsivity or self-control and IGD. Brand’s model [32] proposes that dysfunctional personality traits increase addictiveness to specific internet applications (including internet gaming), through mediation of changes in related cognitions (eg, expectations). Cognition of outcome expectancy regarding addictive behaviors has been shown to partially mediate the associations between impulsivity (a type of dysfunctional personality trait) and addictive behavior (eg, substance use and alcohol drinking) [33,34]. These studies, however, did not look at IGD. Following Brand’s model, this study hence tested whether MCIG would mediate the association between impulsivity and IGD. This contention is indirectly supported by a study that reported a partial mediation of the association between maladaptive personality traits (ie, negative affectivity, detachment, and psychoticism) and IGD via the cognition of expectancies related to using internet gaming to escape from reality [35]. That study, however, did not include other types of MCIG. Furthermore, we did not locate studies that looked at MCIG as a mediator between self-control and IGD. This study thus sought to fill some of deficiencies in the relevant literature.

The mediation between interpersonal influences and IGD via MCIG was also tested in this study. The hypothesis is supported by the theory of triadic influence, which is a framework that incorporates influential factors of addictive behaviors from a number of psychological and sociological theories [36]. It distinguishes between distal or ultimate factors that influence proximal factors of changes in health-related behaviors [37]. According to the theory of triadic influence, interpersonal factors are distal or ultimate variables while cognitive factors related to a certain behavior are proximal variables [36]; the theory suggests that cognitive factors (eg, outcome expectancy) mediate between interpersonal influences and health-related behaviors [38,39]. Such mediations were found for some addictive behaviors (eg, substance use [40], alcohol drinking [41], and internet addiction [29]). One study also reported full and partial mediation of the cognition of positive outcome expectancy of internet gaming between interpersonal influences (peers’ positive attitude toward gaming, intensity of peers’ internet game use, and the frequency of peers’ invitation to play internet games) and IGD among high school students [28]. To our knowledge, however, no study has looked at the mediation effects of other types of MCIG.

We thus investigated the factors of IGD, including impulsivity, self-control, interpersonal influences exerted by parents and peers, and the 3 types of MCIG (perceived rewards, perceived urges, and perceived unwillingness to stop playing) among junior middle school students (grade 7 to 9 of formal education) in 2 populous cities in China. We then tested the mediation effects of MCIG on the associations between impulsivity or self-control and IGD, and between interpersonal influences and IGD, respectively. We hypothesized that the direct and indirect effects would be statistically significant.

## Methods

### Participants and Data Collection

The cross-sectional survey was conducted among secondary school students in Guangzhou and Chengdu in China from October 2018 to December of 2018. The 2 cities are located in southern and southwestern China, which had populations of 14.9 and 16.3 million people in 2018, respectively, and 0.36 and 0.40 million secondary school students in 2018, respectively [42]. Seven junior middle schools (4 out of 409 from Guangzhou and 3 out of 460 from Chengdu) were conveniently selected and participated in the study. All the Grade 7 students (7 years of formal education) in Guangzhou and all the Grade 7 to 9 students in Chengdu were invited to join the survey. The inclusion criteria were those who were full-time Grade 7 students and Grade 7-9 students of the participating schools in Guangzhou and Chengdu, respectively; and those willing to participate in the study. The procedure of data collection was described in a published study that used a subsample of the survey [12], and it is briefly introduced here. Under the supervision of well-trained field workers, students self-administered an anonymous structured questionnaire in the absence of teachers in classroom settings. They were briefed about the objectives of the survey, the return of completed questionnaires implied informed consent, and the students had the right to quit at any time without any negative consequences. No incentives were given to the students. The study was approved by the Survey and Behavioral Research Ethics Committee of the Chinese University of Hong Kong (#SBRE-18-430).

Of the 4350 students invited to participate in the present study, 3147 (72.34%) returned the questionnaire. Among all the returned questionnaires, 66 (2.10%) were removed from data analyses as there were more than 20% of missing data in their response items. Data obtained from the remaining 3081 students (97.90%) were used for data analysis (1126 from Guangzhou [36.55%] and 1955 from Chengdu [63.45%]).

### Measures

#### Background Variables

Background information was collected, including sex, grades, being born in the studied city, whether living with both parents, both father’s and mother’s educational levels (junior middle school or below, senior middle school or equal, or college or above), household income level compared with their classmates (5 points: much higher to much lower), and self-reported academic performance (3 points: above average, average, and below average).

#### IGD Assessment

The 9-item *DSM-5* checklist was used to assess IGD [43]; it recorded the presence of addictive symptoms, including preoccupation, withdrawal, tolerance, inability to control internet gaming, loss of interest in other activities, psychological or social problems, deception, avoidance, and significant loss due to internet gaming. IGD is defined by endorsement of ≥ 5 items (yes-no response options). The Chinese version of *DSM-5* has been validated as having good psychometric properties and diagnostic validity [44,45]. The Cronbach α of the checklist was .79 in the present study.

#### Maladaptive Cognitions Related to Internet Gaming

MCIG was measured by using the Chinese version of the Revised Internet Gaming Cognition Scale (C-RIGCS). It consists of 3 subscales: perceived rewards of internet gaming, perceived urges for playing internet games, and perceived unwillingness to stop playing without completion of gaming tasks. Sample items are “I feel more in control when I play internet games,” “I would feel bad if I was not able to play internet games,” and “I feel uncomfortable thinking about my unfinished goals or objectives in internet games.” The C-RIGCS has been validated in Chinese adolescents and has shown acceptable psychometric properties [12]. The items were rated with 5-point Likert scales (0=never to 4=always), with higher scores indicating higher levels of MCIG. The Cronbach α of the overall scale and its 3 subscales in the present study were .91, .86, .81, and .74, respectively.

#### Impulsivity

Impulsivity was measured by using the 10-item motor impulsiveness subscale of the Barratt Impulsiveness Scale, which indicates the tendency to act on the spur of the moment and with fast reactions [46]. The Chinese version made some cultural adaptations and showed good reliability and construct validity in Chinese adolescents [47]. A sample item is “I do things without thinking.” The items were rated with 5-point Likert scales (1=completely disagree to 5=completely agree), with higher scores indicating higher levels of impulsivity. The Cronbach α of the scale was .91 in this study.

#### Self-Control

Self-control was measured by using the 13-item Brief Self-Control Scale [26], which demonstrated good psychometric properties in Chinese adolescents [48]. A sample item is “I am good at resisting temptation.” The items were rated with 5-point Likert scales (1=never to 5=always), with higher scores indicating higher levels of self-control. The Cronbach α of the scale was .74 in this study.

#### Interpersonal Influences

Interpersonal influences were measured by revising the 6 items that assessed similar tendencies in a previous study [28]. The items included frequency of being invited to play internet games from parents and peers, perceived parents’ and peers’ gaming intensity, and perceived parental influences and peer influences on current internet gaming behavior. Sample items were “How often do your parents invite you to play internet games?”, “How often do your parents play internet games?”, and “To what extent do you think your parents affect your internet gaming behavior?” Confirmatory factor analysis was conducted to examine the 2-factor structure (parental influences and peer influences) of the 6 items, which showed an acceptable goodness of fit (comparative fit index [CFI]=0.97, Tucker-Lewis index [TLI]=0.97, and root mean square error of approximation [RMSEA]=0.09). The items were rated with 4-point Likert scales (1=never/nil to 4=always/severe), with higher scores indicating higher levels of interpersonal influences. The Cronbach α of the overall scale and its 2 subscales in this study were .73, .63 (a Cronbach α >.60 was considered acceptable in previous literature [49,50]), and .71, respectively.

### Statistical Analysis

IGD was used as the binary dependent variable. Univariate logistic regression analysis was conducted to establish the associations between the studied background variables and IGD; crude odds ratios (ORcs) and their respective 95% CIs were derived. Pearson correlation coefficients (r_p_) and Spearman correlation coefficients (r_s_) were derived for continuous and ordinal variables, respectively. The mediation effects were tested by using structural equation modeling (SEM) with weighted least square mean and variance-adjusted estimation. Three latent variables were created: (1) impulsivity or low self-control was derived from the scale scores of impulsivity and self-control (reversed scores), (2) interpersonal influences was derived from the subscale scores of parental influences and peer influences, and (3) maladaptive cognitions was derived from the subscale scores of the 3 types of MCIG. The paths between the 3 latent variables and IGD were fit to test the mediation hypotheses. Recommended goodness-of-fit indicators included CFI ≥0.90, TLI ≥0.90, and RMSEA ≤0.08. The SEM was conducted by using Mplus 7.0; other statistical analyses were performed with SPSS version 21.0 (IBM Corp). Statistical significance was defined as a 2-tailed *P* value <.05.

## Results

### Descriptive Statistics

More than half of the participants were males (1550/3081, 50.31%) and first-year students (1979/3081, 64.23%). More than one-fifth were not born in the city where the study was conducted (691/3081, 22.43%) and did not live with both parents (639/3081, 20.74%). Around one-fifth of the participants’ fathers (594/3081, 19.28%) and mothers (566/3081, 18.37%) had received tertiary education or above; 12.56% (387/3081) self-perceived a lower or much lower household income level than did their classmates, and 19.47% (600/3081) self-reported a below-average academic performance (see Table 1). The mean of impulsivity, self-control, parental influences, and peer influences were 22.9 (SD 7.6, range 10-50), 44.4 (SD 7.6, range 13-65), 4.8 (SD 1.8, range 3-12), and 6.5 (SD 2.1, range 3-12), respectively. Similarly, the mean of the overall C-RIGCS and its 3 subscales were 17.0 (SD 11.6, range 0-60), 7.2 (SD 5.9, range 0-28), 3.7 (SD 3.5, range 0-16), and 6.1 (SD 3.7, range 0-16), respectively.

**Table 1:** Background characteristics of participants (N=3081).

Characteristic			Value, n (%)
**Sex**			
	Female	1525 (49.49)	
	Male	1550 (50.31)	
	Missing data	6 (0.19)	
**Grade**			
	Seven	1979 (64.23)	
	Eight	579 (18.79)	
	Nine	523 (16.98)	
**Study site**			
	Guangzhou	1126 (36.55)	
	Chengdu	1955 (63.45)	
**Born in the city where the study was conducted**			
	Yes	2367 (76.83)	
	No	691 (22.43)	
	Missing data	23 (0.75)	
**Living with both parents**			
	Yes	2382 (77.31)	
	No	639 (20.74)	
	Missing data	60 (1.95)	
**Father’s educational level**			
	Junior middle school or below	1489 (48.33)	
	Senior middle school or equal	831 (26.97)	
	College or above	594 (19.28)	
	Missing data	167 (5.42)	
**Mother’s educational level**			
	Junior middle school or below	1532 (49.72)	
	Senior middle school or equal	803 (26.06)	
	College or above	566 (18.37)	
	Missing data	180 (5.84)	
**Household income level when compared with classmates**			
	Much higher/higher	672 (21.81)	
	Moderate	2002 (64.98)	
	Lower/much lower	387 (12.56)	
	Missing data	20 (0.65)	
**Self-reported academic performance**			
	Above average	1020 (33.11)	
	Average	1348 (43.75)	
	Below average	600 (19.47)	
	Missing data	113 (3.67)	

### Prevalence of IGD

The prevalence of IGD was 13.57% (418/3081; 95% CI 12.4%-14.5%) among all participants. Among those who had played internet games in the past 12 months (2071/3081, 67.22% of all participants), the prevalence of IGD was 17.67% (366/2071; 95% CI 16.0%-19.3%).

### Associations Between Background Variables and IGD

The univariate logistic regression analyses showed that the background variables were all significantly associated with IGD, except for place of birth (whether born in the city where the study was conducted; Table 2). Significant factors included sex (males vs females: ORc=2.80, 95% CI 2.23-3.51), student grade (Grade 8 vs 7: ORc=1.90, 95% CI 1.49-2.44; Grade 9 vs 7: ORc=1.36, 95% CI 1.03-1.80), study site (Chengdu vs Guangzhou: ORc=2.12, 95% CI 1.66-2.69), living arrangement (not living vs living with both parents: ORc=1.54, 95% CI 1.22-1.95), household income level (self-perceived lower or much lower vs higher or much higher than other classmates: ORc=1.64, 95% CI 1.17-2.30), parental education (father’s tertiary vs primary education or below: ORc=0.53, 95% CI 0.39-0.73; mothers’ tertiary vs primary education or below: ORc=0.72, 95% CI 0.53-0.97), and self-reported academic performance (average vs above average: ORc=1.35, 95% CI 1.04-1.76; below average vs above average: ORc=2.53, 95% CI 1.90-3.36).

**Table 2:** Univariate logistic regression analysis on the associations between the studied background variables and internet gaming disorder (N=3081).

Background variables		IGD^a^, n (%)		Association, ORc^b^ (95% CI)
**Sex**				
	Female^c^	119 (7.8)	N/A^d^	
	Male	297 (19.16)	2.80 (2.23-3.51)***	
**Grade**				
	Seven^c^	226 (11.42)	N/A	
	Eight	114 (19.69)	1.90 (1.49-2.44)***	
	Nine	78 (14.91)	1.36 (1.03-1.80)*	
**Study site**				
	Guangzhou^c^	96 (8.53)	N/A	
	Chengdu	322 (16.47)	2.12 (1.66-2.69)***	
**Born in the city where the study was conducted^e^**				
	Yes^c^	308 (13.01)	N/A	
	No	105 (15.2)	1.20 (0.94-1.52)	
**Living with both parents^e^**				
	Yes^c^	294 (12.34)		
	No	114 (17.84)	1.54 (1.22-1.95)***	
**Father’s educational level^e^**				
	Junior middle school or below^c^	227 (15.25)	N/A	
	Senior middle school or equal	109 (13.12)	0.84 (0.66-1.07)	
	College or above	52 (8.75)	0.53 (0.39-0.73)***	
**Mother’s educational level^e^**				
	Junior middle school or below^c^	218 (14.23)	N/A	
	Senior middle school or equal	102 (12.7)	0.88 (0.68-1.13)	
	College or above	60 (10.6)	0.72 (0.53-0.97)*	
**Household income level when compared with classmates^e^**				
	Much higher/higher^c^	87 (12.95)	N/A	
	Moderate	246 (12.29)	0.94 (0.73-1.22)	
	Lower/much lower	76 (19.64)	1.64 (1.17-2.30)**	
**Self-reported academic performance^e^**				
	Above average^c^	98 (9.61)	N/A	
	Average	169 (12.54)	1.35 (1.04-1.76)*	
	Below average	127 (21.17)	2.53 (1.90-3.36)***	

^a^IGD: internet gaming disorder.

^b^ORc: crude odds ratio.

^c^Reference=1.0

^d^N/A: not applicable.

^e^Missing data were excluded from the analysis.

*P<.05.

**P<.01.

***P<.001.

### Correlations Among the Studied Variables

The 3 studied risk factors (impulsivity, parental influences, and peer influences) were all positively correlated with the overall C-RIGCS and its 3 subscales representing MCIG (r_p_ ranged from 0.19 to 0.39; *P*<.001) and IGD (r_s_ ranged from 0.24 to 0.26; *P*<.001), respectively. Self-control was negatively correlated with the overall C-RIGCS and its 3 subscales (r_p_ ranged from –0.45 to –0.27; *P*<.001) and IGD (r_s_=–0.32; *P*<.001), respectively. Besides the above correlations, all the studied factors of IGD were significantly correlated with each other (see Table 3).

**Table 3:** Correlations among impulsivity, self-control, interpersonal influences, maladaptive cognitions, and internet gaming disorder (N=3081)^a^.

Major variable	1	2	3	4	5	6	7	8	9
1. Impulsivity^b^	—^c^								
2. Self-control	0.58*	—							
3. Parental influences^b^	0.26*	0.24*	—						
4. Peer influences^b^	0.24*	0.21*	0.40*	—					
5. Overall maladaptive cognitions^b^	0.37*	0.38*	0.30*	0.39*	—				
6. Perceived rewards of internet gaming^b^	0.31*	0.32*	0.29*	0.37*	0.93*	—			
7. Perceived urges for playing internet games^b^	0.39*	0.45*	0.28*	0.35*	0.85*	0.69*	—		
8. Perceived unwillingness to stop playing without completion of gaming tasks^b^	0.28*	0.27*	0.19*	0.30*	0.85*	0.68*	0.61*	—	
9. IGD^d,e^	0.30*	0.41*	0.37*	0.42*	0.24*	0.26*	0.26*	0.32*	—

^a^Missing data were excluded from the analyses.

^b^Pearson correlation analyses.

^c^Not applicable.

^d^Spearman correlation analyses.

^e^IGD: internet gaming disorder.

*P<.001.

### The SEM Model Testing the Mediation Hypotheses

**Figure 1:**
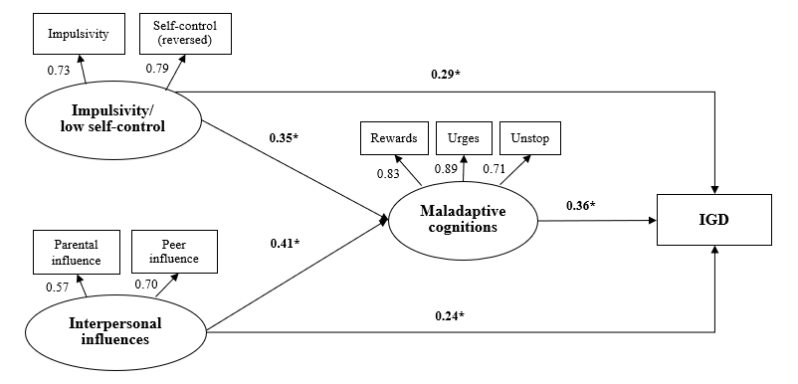
The structural equation model testing the mediation hypotheses. IGD: internet gaming disorder. *P<.001.

Figure 1 presents the SEM model that demonstrated a satisfactory model fit (CFI=0.95, TLI=0.90, and RMSEA=0.08); the factor loadings of the 3 latent variables ranged from 0.57 to 0.89 (all *P*<.001). The findings revealed that maladaptive cognitions partially mediated the association between impulsivity or low self-control and IGD (mediation effect size=30.0%; *P* of Sobel test <.001), and between interpersonal influences and IGD (mediation effect size=37.8%; *P* of Sobel test <.001), respectively. Impulsivity or low self-control (standardized *β*=.29; *P*<.001) and interpersonal influences (standardized *β*=.24; *P*<.001) had significant direct effects on IGD. The standardized *β* values of the other paths are presented in Figure 1.

## Discussion

This study revealed an alarmingly high prevalence of IGD (around 14% among all adolescent participants and 18% among adolescent internet gamers). The prevalence was comparable to the 13% among adolescents of 2 other Chinese cities [51], but much higher than the 2% among Chinese Macau adults [52] and the 3.1% among Australian adolescents [9]. These studies all used *DSM-5* criteria to assess IGD. The high prevalence of IGD signifies the need to regulate internet gaming behaviors and conduct interventions to reduce IGD among adolescents in China, as adolescent IGD has been positively associated with various behavioral problems (eg, aggression and violence [53,54]) and mental health problems (eg, loneliness and depression [4-6]).

In our study, a number of background factors were associated with a higher risk of IGD, including male sex, higher grades, not living with both parents, and self-reported below-average academic performance. First, corroborating previous studies [18,55,56], males were associated with a higher risk of IGD. This finding may be explained by the sex differences in brain responses [57], motives of internet gaming [58], and a less female-friendly gaming environment [59], among other plausible factors. Second, higher grades were positively associated with IGD in this study. Similar findings have been reported [9], but the direction of the association between age and IGD was mixed [18,60]. Future longitudinal studies are warranted to examine the associations between sex, age, grade, and IGD to develop sex- and age-specific interventions to reduce IGD. Furthermore, disadvantaged adolescents, including those of lower socioeconomic status (eg, self-perceived lower household income) and those not living with both parents were more likely than others to have IGD. Similar results were reported in previous longitudinal and cross-sectional studies [61,62]. Disadvantaged adolescents might have fewer resources for leisure activities and less parental monitoring over internet use, which may lead to more intensive internet gaming and higher risk of developing IGD [61]. A negative association between academic performance and IGD was also reported in our study, which was consistent with previous literature [18,63,64]. It is worth noting that the relationship between academic performance and IGD may be bidirectional. Frustration over poor academic performance may lead to problematic gaming as a means of escape and maladaptive coping [65], which has been associated with IGD [66]. Conversely, IGD may lead to poor academic performance [5,67]. The causal direction needs to be confirmed by longitudinal studies.

To facilitate the design of effective interventions for reducing IGD, it is important to understand the mechanisms (mediation) underlying the associations between risk or protective factors of IGD and IGD. The findings reveal that some intrapersonal (impulsivity) and interpersonal (interpersonal influences) risk factors may elevate the levels of the 3 types of MCIG, which may in turn increase the risk of developing IGD. Furthermore, self-control may reduce MCIG, which may increase IGD (a partial mediation effect). Nonetheless, the presence of significant direct effects between impulsivity, self-control, and interpersonal influences and IGD imply the existence of other unstudied mediators. For instance, coping is a potential mediator, as Brand’s model postulates dysfunctional copying strategies mediate between personality traits and problematic use of the internet (including IGD) [32], while impulsivity can be considered a personality trait. Interpersonal influences may also strengthen the subjective norms of internet gaming (ie, significant others’ support for internet gaming), which is a construct of the theory of planned behaviors [68]; these subjective norms are expected to be associated with MCIG. Future studies should look at other mediators.

The observed mediation effects suggest that modifications of the 3 types of MCIG can potentially reduce the harmful impacts of impulsivity or interpersonal influences on IGD and increase the protective effect of self-control against IGD. Targeted interventions to improve MCIG may include training to increase awareness of the 3 types of MCIG and skills to perform related cognitive reconstructions [69], provision of alternative sources of rewards (eg, outdoors activities) to reduce perceived rewards of internet gaming, removal of sources of temptations and stimuli (eg, gaming devices), and introduction of distraction skills to reduce perceived urges or unwillingness to stop playing internet games [70,71].

There are also plausible explanations for why impulsivity was positively associated with the 3 types of MCIG. First, impulsivity may increase reward sensitivity that enhances adolescents’ drives to seeking more rewards from addictive behaviors [72,73]; impulsive adolescents may thus possess a higher reward drive and perceive more rewards from internet gaming. Second, impulsive adolescents may be more responsive to cues of internet gaming and thus hold stronger urges for playing. Third, impulsivity in general may reduce impulse inhibitions against addictive behaviors, even in the presence of negative consequences [74-76]; adolescents with weakened inhibitions of gaming impulses may thus be less able to resist stimuli inductive to playing internet games and may perceive stronger unwillingness to stop playing. Self-control was negatively associated with the 3 types of MCIG, possibly because of the negative association between impulsivity and self-control [22-25], but other reasons may also apply. Interventions for modifying impulsivity and self-control may remove temptations and strengthen self-efficacy in regulating impulses [77]). In particular, the if-then planning intervention that specifies when, where, and how to regulate impulses is potentially useful [78]; a review reported that this type of intervention showed efficacy in reducing addictive behaviors (eg, in binge drinking and cigarette smoking) [79].

This study also found positive associations between interpersonal influences and MCIG. Having significant others who are frequent internet gamers was positively associated with MCIG. The SCT suggests that, through reciprocal determinism and observational learning, social interactions may influence both adolescents’ attitudes and behavior [31]. It is likely that adolescents’ parents or peers who play internet games frequently may also perceive higher levels of MCIG (eg, the 3 types of MCIG). Adolescents’ maladaptive cognitions may be influenced by those of their parents via multiple means, such as social learning, subjective norms, and reinforcement. Furthermore, peers influence each other to form common perceptions regarding particular behaviors [80]. Future confirmation is needed. Family-based interventions for reducing IGD are potentially useful. A psychoeducation on maladaptive and adaptive use related to internet games showed preliminary effectiveness [81]. Another intervention that involved parental monitoring on adolescents’ self-regulation practice and gaming behaviors showed short-term (3 month) efficacy in improving attitude, knowledge, self-regulation, and IGD among adolescents [82]. Although a meta-analysis showed that peer-led interventions were efficacious in reducing tobacco, alcohol, and substance use among adolescents [83], our literature search did not locate similar interventions for IGD. Future evidence-based interventions are warranted.

This study has several limitations. First, reporting bias, such as recall bias and social desirability bias, might have been introduced. Second, as the participating schools were selected based on convenience, there might have been selection bias, and generalization of the study results should be done cautiously. Third, the prevalence of IGD assessed by using the *DSM-5* checklist might have been overestimated compared with that based on the *ICD-11* criteria [84]. Fourth, we were unable to make causal inferences due to the cross-sectional nature of this study. Fifth, the revised assessment tool of interpersonal influences has not been validated although we conducted confirmatory factor analysis to test its 2-factor structure, which showed an acceptable goodness of fit. Finally, this study only investigated internet gaming but not offline video games, while both online and offline video games are included in the *ICD-11* under the category of gaming disorder, as the 2 types of gaming disorder differ in etiology, epidemiology, and treatment.

In conclusion, MCIG partially mediated the associations between impulsivity, self-control, or interpersonal influences and IGD. Modifications of the 3 types of MCIG may effectively reduce the harmful impacts of impulsivity or interpersonal influences on IGD and increase the protective effect of self-control against IGD. Future longitudinal studies are warranted to verify these findings and explore other potential mediators.

## Abbreviations

CFI: comparative fit index
C-RIGCS: The Chinese version of the Revised internet Gaming Cognition Scale
*DSM-5*: *Diagnostic and Statistical Manual of Mental Disorders, fifth edition*
*ICD-11*: *International Classification of Disease, eleventh revision*
IGD: internet gaming disorder
MCIG: maladaptive cognitions related to internet gaming
ORc: crude odds ratio
RMSEA: root mean square error of approximation
SCT: social cognitive theory
SEM: structural equation modeling
TLI: Tucker-Lewis index
WHO: World Health Organization
